# Cross‐sectional analysis of healthy individuals across decades: Aging signatures across multiple physiological compartments

**DOI:** 10.1111/acel.13902

**Published:** 2023-06-23

**Authors:** Ruin Moaddel, Ceereena Ubaida‐Mohien, Toshiko Tanaka, Qu Tian, Julián Candia, Ann Zenobia Moore, Jacqueline Lovett, Giovanna Fantoni, Nader Shehadeh, Lisa Turek, Victoria Collingham, Mary Kaileh, Chee W. Chia, Ranjan Sen, Josephine M. Egan, Luigi Ferrucci

**Affiliations:** ^1^ Biomedical Research Centre National Institute on Aging, NIH Baltimore Maryland USA

**Keywords:** aging, inflammation, kidney function, mitochondrial health, muscle metabolomics, plasma metabolomics, senescence, urine metabolomics

## Abstract

The study of age‐related biomarkers from different biofluids and tissues within the same individual might provide a more comprehensive understanding of age‐related changes within and between compartments as these changes are likely highly interconnected. Understanding age‐related differences by compartments may shed light on the mechanism of their reciprocal interactions, which may contribute to the phenotypic manifestations of aging. To study such possible interactions, we carried out a targeted metabolomic analysis of plasma, skeletal muscle, and urine collected from healthy participants, age 22–92 years, and identified 92, 34, and 35 age‐associated metabolites, respectively. The metabolic pathways that were identified across compartments included inflammation and cellular senescence, microbial metabolism, mitochondrial health, sphingolipid metabolism, lysosomal membrane permeabilization, vascular aging, and kidney function.

## Graphical abstract

A targeted metabolomic analysis of plasma, skeletal muscle, and urine from healthy participants was carried out to compare and contrast metabolite changes with aging across and within compartments. The metabolic pathways that were identified included inflammation and cellular senescence, microbial metabolism, mitochondrial health, sphingolipid metabolism, lysosomal membrane permeabilization, vascular aging, and kidney function.

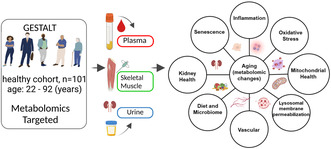

## 1 INTRODUCTION

Human studies make use of biomarker changes in fluids and tissues, such as blood, skeletal muscle, and urine, that occur during the aging process to infer biological changes due to aging per se (Johnson et al., 2019; Teruya et al., 2020; Ubaida‐Mohien, Lyashkov, et al., 2019). These studies are made even more complex by the difficulty in distinguishing between changes compensatory to the emergence of pathology and those that simply reflect aging itself (Cohen et al., 2022). In addition, findings from studies that investigated age‐related changes in the metabolome that occurred in just one compartment, such as plasma or serum, may not necessarily translate to other biological fluids or tissues. We hypothesized that simultaneously investigating age‐related differences in various fluid and tissue compartments may shed more light on the underlying mechanisms that drive changes in the metabolome with age and that ultimately contribute to the phenotypic manifestations of aging. Here, we report the results of a comprehensive profiling of age‐related metabolomic changes across three compartments simultaneously: the circulatory system (plasma), the excretory system (urine), and a solid organ (skeletal muscle). To minimize the interference of changes in metabolites reactive to pathology, we enrolled in this study individuals that were healthy based on a comprehensive clinical evaluation performed by trained health professionals. To reduce variability that can result from different quantification methods, the same targeted metabolomic platform was used for all compartments. Herein, we carry out a cross‐sectional analysis of ‘healthy’ individuals who were free from disease to formulate hypotheses based on the metabolic exchanges that occur between different compartments with aging.

## 2 RESULTS

### 2.1 Analysis of healthy human aging in plasma, skeletal muscle and urine and plasma validation

**FIGURE 1:**
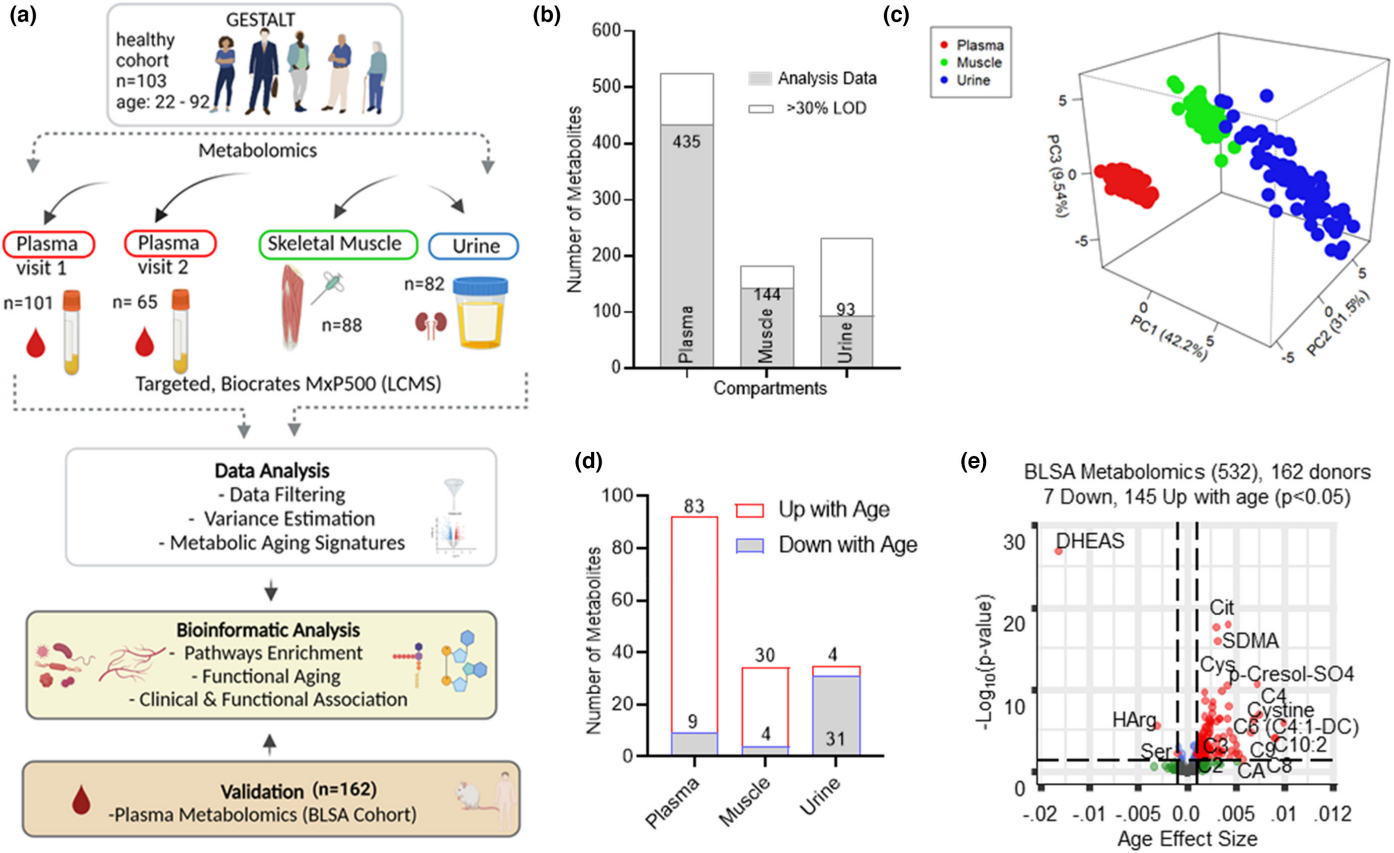
Analysis of healthy human aging in plasma, skeletal muscle and urine and plasma validation. (a) Overview of metabolomic aging signature workflow. (b) Number of metabolites quantified from different compartments. Missing data (>30% LOD) are further analyzed for age‐associated missing specificity (see methods). (c) Principal component analysis of all metabolites quantified from participants from all three compartments. Compartments are color‐coded. (d) Number of upregulated and downregulated age‐associated metabolites from plasma, muscle, and urine. (e) GESTALT plasma metabolite validation using an independent, healthy aging cohort (BLSA). Volcano plot of all metabolites quantified from 162 BLSA participants, age ranging from 22 to 97 years. Of the 532 BLSA metabolites, 152 age‐associated metabolites are shown above the dotted line (*p* < 0.05). Metabolite significance with age is plotted on the *y*‐axis, metabolite‐ageassociation is plotted on *x*‐axis, and positive age effect size shows metabolites association higher in older age and negative age effect size shows association lower in older age.

We conducted a targeted metabolomic study from ‘healthy’ participants (Tanaka et al., 2018) of the Genetic and Epigenetic Study of Aging Laboratory Testing (GESTALT, age‐range 22–92 years) in plasma sample (baseline *n* = 101 and 2‐year follow‐up *n* = 65), skeletal muscle biopsy specimens (baseline *n* = 88), and urine (baseline *n* = 82). Participants enrolled in GESTALT had no medical history of cardiovascular, pulmonary, gastrointestinal, autoimmune, or metabolic diseases, had no history of cancer within the preceding 10 years. Main demographics of the study are summarized in Table 1, and the study design is illustrated in Figure 1a. Of the 473 measured metabolites, 435, 144, and 93 metabolites were quantifiable in plasma, skeletal muscle, and urine, respectively (Figure 1b, Table S1). Plasma, muscle and urine metabolites had a clear separation between compartments, with the first three principal components explaining 83.2% of the variance (Figure 1c), suggesting differential expression of a group of metabolites for a specific compartment. Moreover, the 3D PCA analysis demonstrates that different compartments have different abundance profiles with respect to metabolites and suggests that the compartment effect is more pronounced than the aging effect (Figure S1). The top metabolites common across all three compartments contributing to the PCA (PC1, PC2, and PC3 coefficients) are reported in Table S2. Linear regression model adjusted for sex, race, and body mass index (BMI) were performed to identify metabolites that were significantly associated with age. Of the quantifiable metabolites in each compartment, there were 92 (21.15%), 34 (23.6%), and 35 (37.6%) metabolites associated with age (*p* < 0.05) in plasma, skeletal muscle, and urine, respectively (Figure 1d, Table S3). In our study urine samples were not normalized to creatinine, as it is well known that aging is associated with a decline in glomerular filtration rate (GFR; O'Sullivan et al., 2017). This is consistent with other studies that suggested normalization to urinary creatinine levels may result in an under/over estimation of biomarkers based on the clinical context (Waikar et al., 2010). However, adjustment to urinary and plasma cystatin C levels, the majority of identified age‐associated metabolites were similar across groups (Table S4A,B, respectively). The low coverage of the metabolites in urine and muscle compared to plasma likely reflects low concentrations of those metabolites in those compartments. For the 419 common plasma metabolites assessed both at baseline and 2‐year follow‐up, there was a modest significant correlation of the β coefficient for age (*r* = 0.31, *p* = <3.2e‐35; Figure S2A). When examining 92 age‐associated metabolites, the correlation between the age beta coefficient was highly significant (*r* = 0.79, *p* = 2.2e‐16; Figure S2B), suggesting the reliability of our method. 91 of them changed with age in the same direction, among which 27 were statistically significant. The modest sample size and limited follow‐up time span may have affected the statistical power.

**TABLE 1:** Demographics of the participants of the genetic and epigenetic study of aging laboratory testing (GESTALT, age‐range 22–92 years) in plasma sample, skeletal muscle biopsy specimens, and urine.

Matrix	Phenotype			Age groups (*n*)				
	*n*	BMI	Sex (male%)	20–34	35–49	50–64	65–79	80+
Plasma	101	25.59	55	23	20	18	28	12
Skeletal muscle	88	25.77	57	21	18	16	23	10
Urine	82	25.44	52	17	18	17	20	10

We next replicated the baseline plasma findings in 162 participants of the Baltimore longitudinal study of aging (BLSA; Table S5A) that were selected to have the same healthy characteristics of the GESTALT study (see methods section) and using the same metabolomic platform as in GESTALT (Figure 1e). Again, the age β coefficients obtained in the GESTALT and BLSA cohorts were highly correlated (*r* = 0.64; *p* < 2.2e‐16; Figure S3A) suggesting that age‐related differences in metabolites are similar across different studies (Figure 1e, Figure S3A). When the analysis was limited to the 92 metabolites that were significantly age‐associated in the GESTALT study, the age β coefficient correlation was even stronger (*r* = 0.80, *p* = <2.2e‐16) (Figure S3B). There were 57 common age‐associated metabolites in the GESTALT and BLSA cohorts (Figure S3C). While there was a strong agreement of the results between BLSA and GESTALT cohorts there were also observations unique to each cohort. While we cannot fully exclude a role of chance, differences between the two cohorts may explain some of the variation in the results. While the inclusion criteria for ‘healthy’ in BLSA and GESTALT are exactly the same in both studies, once participants are enrolled, they remain in the study regardless of changes in health or functional status. The plasma samples from GESTALT were collected at enrollment while the BLSA plasma samples assessed for this study were collected many years after enrollment. Further, the distribution of participants across age is relatively equal in GESTALT while the BLSA sample has a higher proportion of older participants (>80 years of age; Table S5B).

### 2.2 Differences in the plasma metabolome with aging

**FIGURE 2:**
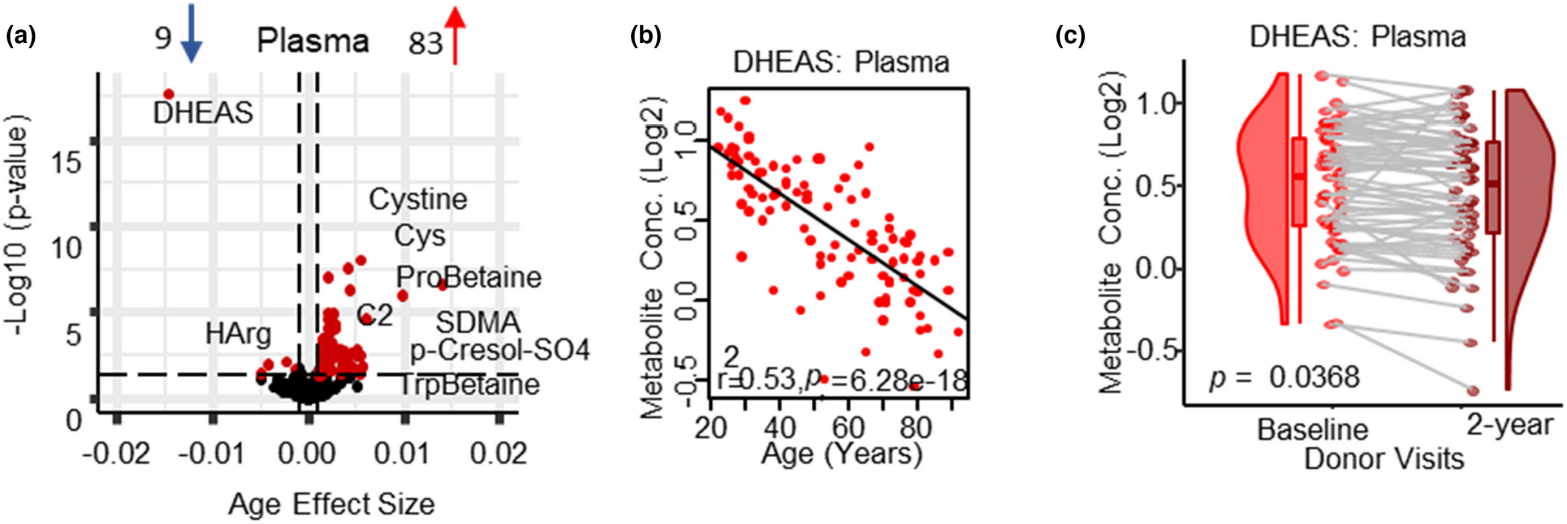
Changes in the plasma metabolome with aging. (a) Volcano plot shows age association of plasma metabolites, Significant metabolites (*p* < 0.05) are represented as red circles and the most significant metabolites are labeled. The number of upregulated and downregulated metabolites are shown on the volcano plot title. Age beta (*x*‐axis) represents the metabolites change over a year of age, either positively or negatively correlated with human age. Positive (83 metabolites) or negative (9 metabolites) age association is shown as red and blue on the top. (b). Top age‐associated metabolite is shown (DHEAS) (c) and a negative correlation of DHEAS with age is validated in plasma between baseline and the 2‐year follow‐up.

Of the 435 plasma metabolites analyzed, 92 metabolites (83 increased, 9 decreased) were age‐associated (Figure 2a), of which 70 metabolites (67 increased, 3 decreased) were only age‐associated in plasma (Figure 2a, Table S3; Figure S4). In addition, 17 metabolism markers, calculated from sums and/or ratios of metabolites (www.biocrates.com), were assessed (Table S6) and of these, eight metabolism indicators were age‐associated in plasma, with five decreasing (Arginine (Arg)/symmetric dimethylarginine (SDMA) and Arg/asymmetric dimethylarginine (ADMA), global arginine bioavailability ratio (GABR), proline (Pro)/citrulline (Cit), serine (Ser)/glycine (Gly)) and two increasing (kynurenine (Kyn)/tryptophan (Trp) and cystine/cysteine (Cys)). The ester of dehydroepiandrosterone (DHEAS), which is synthesized in adrenal cortex, was the top age‐associated plasma metabolite and was significantly lower in older participants (Figure 2b). This decline of DHEAS with aging was previously reported (Orentreich et al., 1992), and was confirmed both cross‐sectionally and longitudinally (over 2‐year follow‐up) (Figure 2c) with a 1.07% annual decline in plasma, which is lower than ~2% annual decline reported in the literature (Sahu et al., 2020), likely indicative of our healthy cohort. Cystine was the second most significantly age‐associated metabolite, followed by phosphatidyl choline (PC) diacyl (aa) C40:6, Cys and Pro betaine. Interestingly, several of the age‐associated metabolites found in this study (Table S3) are considered biomarkers of oxidative stress and inflammation, Arg bioavailability, one‐carbon metabolism, perturbed sphingolipid metabolism and healthy diet (Table S7). Of the measured bile acids, only glycolithocholic acid (GLCA), an agonist for the TGR5 receptor that is a metabolic regulator involved in energy and glucose metabolism (Klindt et al., 2019), was significantly higher at older age.

Consistent with findings reported above, the redox potential biomarker (Cystine/Cys ratio) was the second strongest age‐associated metabolism indicator by significance (Table S6), suggesting an increase in oxidative stress with age, and in agreement with published data that a shift of the disulfide/thiol toward a redox state occurs between the third and tenth decade of life (Hack et al., 1998). The increase in the cystine/cys ratio has also been associated with an increase in IL‐1β and TNF‐α, both pro‐inflammatory cytokines, in the plasma of healthy adults (Iyer et al., 2009). Consistent with this interpretation we observed an increase in Kyn and the immune checkpoint protein, indoleamine 2,3‐dioxygenase (IDO) activity (Kyn/Trp ratio). IDO activity is upregulated by pro‐inflammatory cytokines (Moaddel et al., 2022), is high in nonagenarians and has been found to be associated with higher mortality and frailty in older persons (Marttila et al., 2011). The activation of aryl hydrocarbon receptor (Ahr), via Kyn, can also stimulate expression of IDO1, and can modulate levels of ROS, resulting in a positive feedback loop potentially linking inflammation with ROS production (Kaiser et al., 2020).

A decrease in the bioavailability of Arg was also observed in plasma (Table S7). Arg is the critical amino acid substrate necessary for nitric oxide (NO) production and low NO production has been associated with higher pro‐inflammatory cytokines (Moaddel et al., 2022). NO is the most powerful endogenous gaseous vasodilator, and additionally it stimulates mitochondrial function and mitogenesis and higher levels are considered protective against cardiovascular disease. Reduced bioavailability of Arg may contribute to the increased incidence and prevalence of cardiovascular diseases with aging. Consistent with this interpretation, we also found several additional indicators of lower NO production in plasma, including a decline in HArg (competitive NOS substrate) and an increase of endogenous NOS inhibitors (ADMA, SDMA) with age, as well as age‐associated decreases of Arg/ADMA and Arg/SDMA ratios (Tables S3 and S6).

The majority of plasma age‐associated metabolites (65 out of 92) were lipid species, including 23 PCs and 24 ceramides (Cer; Table S3) predominantly increasing with age, with the exception of PC acyl‐alkly (ae) C34:3, lysophosphatidylcholine (LPC) 16:0 and triacylglycerol (TG) (22:4_34:2) that declined with age. These findings are consistent with previous studies that reported that healthy subjects had higher plasma levels of PCs and SMs compared to young subjects (Trabado et al., 2017). The decrease of LPC 16:0 and trending decrease of LPC 18:2 (*p* = 0.063) can affect mitochondrial membrane architecture and function as LPC is a precursor to cardiolipin via lysophosphatidic acid and may be a limiting factor for the biogenesis of new mitochondria, which may both contribute to lower oxidative capacity and increase inflammation with aging (Semba et al., 2019). Previously we have demonstrated that changes in specific LPCs are associated with concurrent decline in skeletal muscle mitochondrial function (Tian et al., 2022). An age‐associated increase in 24 ceramide species in plasma includes 8 ceramides, 6 hexosyl ceramides (HexCer), and 10 sphingomyelins (SMs), indicating perturbed sphingolipid metabolism. Five of the age‐associated ceramides contained nervonic acid (NA; Table S3), a fatty acid that is essential for growth and maintenance of brain integrity and participates in the repair of damaged neurons (Lewkowicz et al., 2019). Increasing circulating levels of NA containing ceramides may be due to myelin degeneration with age and/or partaking of a diet rich in NA (swordfish, salmon, sesame seeds, quinoa), as it was shown that fish oil (rich in EPA, DHA, and NA) improves the ability of mature oligodendrocytes to synthesize myelin proteins as well as SM (Lewkowicz et al., 2019). Interestingly, several of the age‐associated ceramides, SMs, cholesterol esters (CE) and TGs contained an Ω‐3 fatty acid (DHA, DPA, EPA), palmitic acid, and/or linoleic acid fatty acids.

### 2.3 Differences in the skeletal muscle metabolome with aging

**FIGURE 3:**
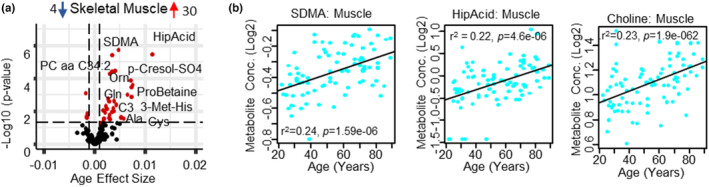
Changes in the skeletal muscle metabolome with aging. (a) Volcano plot shows age association of skeletal muscle metabolites. Significant metabolites (*p* < 0.05) are represented as red circles and the most significant metabolites are labeled. The number of upregulated (Kochlik et al., 2019) and downregulated (Cohen et al., 2022) metabolites are shown on the volcano plot title. Age beta (*x*‐axis) represents the metabolites change over a year of age, either positively or negatively correlated with human age. (b). Top age associated SDMA, hippuric acid, and choline metabolite in skeletal muscle, log2 metabolite concentration is shown on *y*‐axis and age on *x*‐axis.

Thirty‐four metabolites were age‐associated in muscle (30 increased, 4 decreased), of which 14 metabolites (11 increased, 3 decreased) were muscle compartment specific (Figure 3a, Table S3, Figure S4). Choline was the top age‐associated metabolite, followed by hippuric acid, SDMA, and Orn (Figure 3b). In addition, four metabolism indicators were age‐associated of which Arg/SDMA (biomarker of Type II protein arginine methyltransferases activity) and Arg/Orn (biomarker of arginase activity) were lower with older age, while Orn/Cit (biomarker of ornithine transcarbamoylase activity) were higher. The majority of age‐associated metabolites and metabolism indicators were related to mitochondrial health, Arg bioavailability, and one‐carbon metabolism (Table S7). Because skeletal muscle is a highly energetically demanding tissue, it is not surprising that age‐related differences of metabolites reflecting mitochondrial health were more evident compared to other tissues (Smuts et al., 2013).

Choline plays several important roles in skeletal muscle processes, including fat and protein metabolism, inflammation, and autophagy. Moreover, it contributes to muscle growth and function, ion movements and mechanisms of muscle contraction because it is a precursor of the neurotransmitter acetylcholine (Moretti et al., 2020). It is an essential micronutrient that plays a pivotal role in several metabolic pathways contributing to liver, neurological, and hematological homeostasis (Moretti et al., 2020) and as a methyl‐group donor, influences protein homeostasis by increasing synthesis and reducing breakdown (Moretti et al., 2020). Choline being the most significant age‐associated metabolite in muscle may reflect a continued functional maintenance and repair mechanism in healthy aging. Increased choline levels in muscle can positively contribute to mitochondrial energy metabolism and lipid metabolism by decreasing fatty acid synthesis and stimulating PC synthesis (Moretti et al., 2020). Accordingly, in this study, 9 of the 13 age‐associated PCs increased with aging, of which 9 (6 increasing/3 decreasing) were compartment specific. Choline is also a precursor for TMAO, which is generated via gut microbial metabolism (Bennett et al., 2013), where the production of TMAO is heavily influenced by the composition of the gut microbiome. TMAO levels reflect a diet rich in eggs, meat, and seafood, with fish consumption resulting in a more pronounced increase (Cho et al., 2017). In muscle, TMAO increased with age, and trended upwards with age in plasma (*p* = 0.055), consistent with the age‐associated increase of choline in plasma and muscle. The elevated levels of hippuric acid can likely be explained by a healthy diet rich in fruits and vegetables in this population and is consistent with its positive health profile since low hippuric acid levels have been previously associated with frailty (Brunelli et al., 2021). Other differences that are also consistent with the healthy characteristics of this population include the age‐associated increases of Arg, Lys, Gln, and GABA (Mirzaei et al., 2014). The maintenance of Arg and Lys levels in skeletal muscle may help minimize biological aging and frailty as Arg provides a substrate for NO synthesis, is an important amino acid for muscle protein synthesis, and contributes to quenching oxidative stress, especially after exercise (Moaddel et al., 2022; Tokarz et al., 2021). In agreement with this interpretation, the members of the polyamine pathway, Orn and putrescine were significantly higher at older age. Of note, an increase in the putrescine/spermidine ratio (*p* = 6.6 e‐7) (biomarker of spermidine synthase) was also observed in muscle. These data suggest that there is increased metabolism of Arg through the polyamine pathway and that the increase in spermidine levels may result from a lower spermine synthase activity and/or higher spermine oxidase activity, consistent with previous reports of lower enzymatic activity in the polyamine synthesis pathways with age (Soda, 2022).

Several age‐associated differences in metabolites suggest age‐associated declines of mitochondrial function even in this very healthy population. These differences include an age‐associated increase of alanine and acyl carnitines, and a trending increase of lactic acid (*p* = 0.074), possibly indicating the utilization of anaerobic glycolysis, which often occurs when oxidative phosphorylation is critically low. The increase in carnitine, C3 (propionyl carnitine), and C5‐OH (C3‐DC‐M), suggests impaired skeletal muscle oxidative capacity and inefficient or incomplete fatty acid β‐oxidation, resulting in an increase in ROS (Aguer et al., 2015). Interestingly, we did not detect branch chain amino acids (BCAA) differences with age in skeletal muscle. The lack of change in BCAA metabolites are consistent with a previous skeletal muscle proteomics study in the same subjects, where changes in key proteins involved in this pathway (BCKDHB, BCKDHA, BCKDK, and RPCK1, for example) were not age‐associated (Ubaida‐Mohien, Gonzalez‐Freire, et al., 2019; Ubaida‐Mohien, Lyashkov, et al., 2019). The increase of several amino acids likely results from decreased protein synthesis (Tokarz et al., 2021) and increases in protein breakdown with age (Kochlik et al., 2019). The increase of 3‐methyl histidine is suggestive of lower protein synthesis with older age, but the lack of an age association of 3‐methyl histidine in the plasma compartment (*β* = 0.0027, *p* = 0.24) indicates the difference is confined to the muscle compartment and is indicative of a healthier cohort, as circulating plasma 3‐methyl histidine levels has been identified as a potential biomarker of frailty (Kochlik et al., 2019).

### 2.4 Differences in the urine metabolome associated with age

**FIGURE 4:**
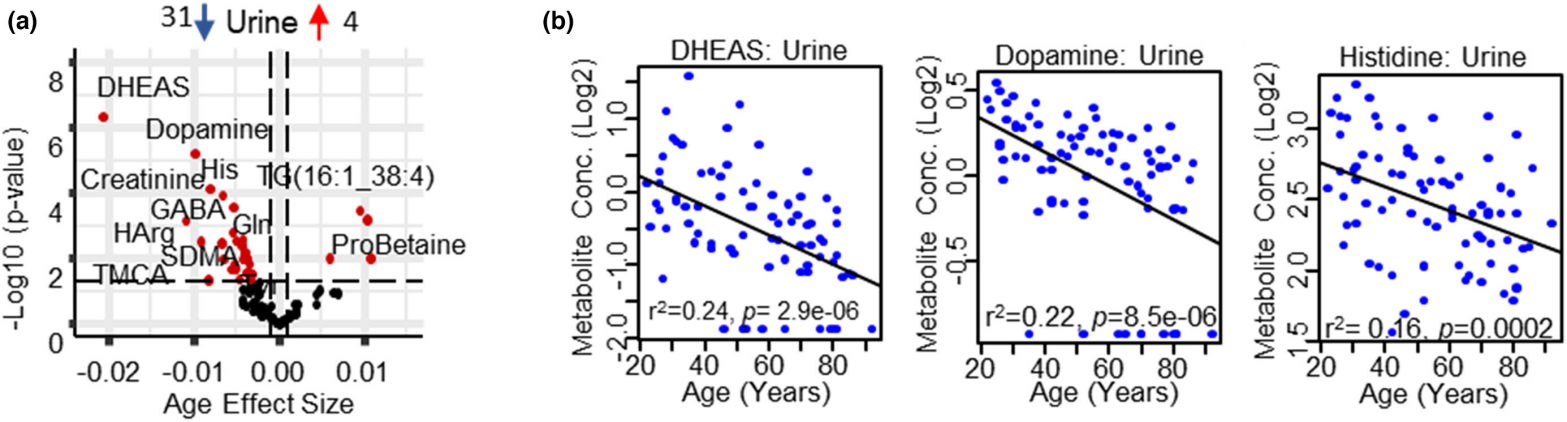
Changes in the urine metabolome associated with age. (a) Volcano plot shows age association of urine metabolites. Significant metabolites (*p* < 0.05) are represented as red circles and the most significant metabolites are labeled. The number of upregulated (Cohen et al., 2022) and downregulated (Sarkar et al., 2017) metabolites are shown on the volcano plot title. Age beta (*x*‐axis) represents the metabolites change over a year of age, either positively or negatively correlated with human age. (b) Top age‐associated DHEAS, dopamine and histidine metabolites in urine, log2 metabolite concentration is shown on y‐axis and age on *x*‐axis.

Contrary to the other compartments, of the 35 age‐associated metabolites in the urine, the majority were lower in older age (31 metabolites), with only proline betaine and three TGs increasing with age. Only 4 PCs were detected in urine and all were negatively age‐associated with PC ae C38:6 trending (*p* = 0.089). DHEAS was the most strongly age‐associated metabolite with a 1.51% annual decline in urine, in line with lower adrenal DHEAS synthesis with age, followed by dopamine, histidine (His), serotonin, and creatinine (Figure 4b). Seventeen age‐associated metabolites in urine were compartment specific (3 increased, 14 decreased) (Figure S4), including the age‐associated decrease of trans‐4‐hydroxyproline, a major amino acid‐related metabolite of collagen proteins (Sarkar et al., 2017). Interestingly, the age‐associated metabolites rate of decline can be clustered into three groups with the largest (excluding DHEAS) from 5 metabolites (HArg, carnosine, His, TMCA, dopamine) with an average *β* = −0.009 ± 0.001, a second group of 10 metabolites with an average *β* = −0.006 ± 0.0007 and the third group of 15 metabolites with an average *β* = −0.004 ± 0.0004. There are two major mechanisms by which metabolites can change in urine: a decrease in plasma levels or reduced clearance with aging. Of interest, the age‐associated metabolites in urine that had the largest decreasing rates (*β* ~ −0.0093) were also decreasing in plasma, albeit not all significant, suggesting those metabolites result from decreased circulating levels in plasma. Conversely, those metabolites that had the smallest annual rate of decrease in urine (*β* ~ −0.004) had several metabolites that increased with age in plasma, suggesting reduced clearance with aging. Interestingly, almost all the age‐associated amino acids in urine had ~0.27% annual decline ranging from glutamate (Glu)—one of the most abundant amino acids—decreasing by 0.23% per year, to Gln decreasing by 0.32% per year. Several amino acids that were trending down with age (alanine, Arg, methionine, Gly) also declined at a similar rate with only His at a steeper rate, with 0.59% annual decline. Several His‐related metabolites, including 1‐methyl histidine which had a 0.29% per year decline in urine and carnosine had a 0.66% annual decline, similar to His decline (0.59%): histamine was not quantifiable and anserine, β‐alanine and 3‐methyl histidine were not age‐associated in urine. Lower levels of His‐related metabolites (carnosine, for example) may be an indication of compensation and/or increased re‐absorption during healthy aging, as His and carnosine have beneficial roles as anti‐oxidant and anti‐inflammatory factors because their imidazole rings scavenge ROS during inflammation (Peterson et al., 1998; Table S7). However, as carnosine was not detected in any other compartment and anserine was measured only in muscle and urine, it is difficult to speculate on the mechanisms involved. Four metabolism indicators were age‐associated with an increase in Cystine/Cys, and Kyn/Trp and a decrease of BCAA and aromatic amino acids with age (Table S7). Lower levels of the majority of age‐associated metabolites including uremic toxins (ADMA, SDMA, creatinine) with age is likely due to a decline of GFR that inevitably occurs with aging even in the healthiest individuals (O'Sullivan et al., 2017).

### 2.5 Common aging metabolomic signatures in plasma, skeletal muscle and urine

**FIGURE 5:**
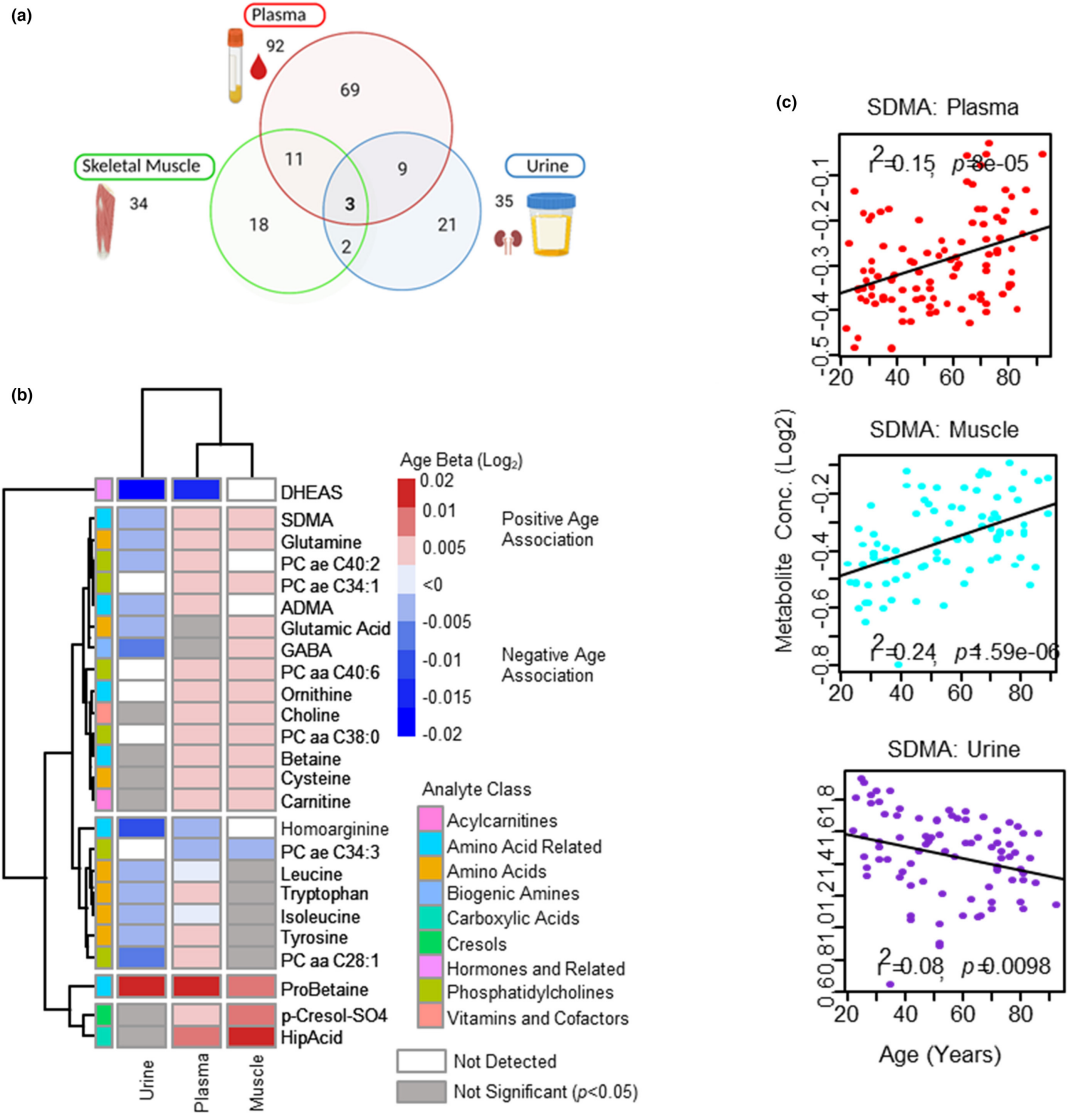
Common aging metabolomic signatures in plasma, skeletal muscle, and urine. (a) Venn diagram of age‐associated metabolites from plasma, muscle, and urine. Shared metabolites are shown in the intersections. (b) Top age‐associated metabolites are shown across different compartments. Positive or negative age association is shown as red and blue with intensity represented larger β, and gray represents nonsignificant metabolites. White color is coded for non‐detected metabolites *(<LOD*). Metabolite analyte classes are color‐coded. (c) Age association of SDMA metabolite from different compartments, log2 metabolite concentration is shown on y‐axis and age on x‐axis, each circle is a participant.

In the metabolomic analysis, there were 25 age‐associated metabolites across compartments, with three metabolites age‐associated in all three compartments (plasma, muscle and urine) and 22 age‐associated metabolites in two compartments (Figure 5a). The three age‐associated metabolites in all three compartments are proline betaine, SDMA and Gln. The increase in proline betaine likely reflects the healthy characteristics of the participants, as it was identified as a biomarker of a healthy diet in a multi‐population epidemiological study reflective of citrus consumption (Heinzmann et al., 2010). SDMA and Gln increased in plasma and muscle, while decreasing in urine (Figure 5b). SDMA is removed almost exclusively via renal excretion and the age‐associated increase likely results from reduced kidney filtration with age. Gln has been previously shown to be elevated in centenarians (Montoliu et al., 2014) and is an important energy source because it enters the TCA cycle for ATP production (Mirzaei et al., 2014) and its increase is therefore consistent with healthy aging, as higher Gln levels in muscle and serum with age could indicate reduced fatty acid oxidation and decreased inflammation (Mirzaei et al., 2014). However, we cannot exclude the possibility that higher Gln levels can also result from decreased excretion.

Of the 22 age‐associated metabolites quantified in two compartments, 9 were age‐associated in plasma and urine, 11 in plasma and muscle and 2 in muscle and urine, with DHEAS, choline and GABA the top age‐associated metabolites across two compartments, respectively (Figure 5b). Of the 9 age‐associated metabolites in plasma and urine, five metabolites (DHEAS, HArg, isoleucine, leucine, Trp) were lower with age in both plasma and urine suggesting that the decline in urine reflects circulating levels in plasma. A decline in kidney excretion capacity with age was also observed as 6 metabolites that increased with age in plasma (ADMA, SDMA, Gln, tyrosine, PC aa C28:1, PC ae C40:2) and had a corresponding decrease in urine (Figure 5b) with Gln and SDMA also increasing in muscle. While a decline in kidney excretion capacity is expected with age, the limited number of age‐associated metabolites in urine that increased in plasma may reflect the healthy status of the participants and result from a slower rate of decline in kidney excretion capacity with age and/or reabsorption of metabolites in the proximal tubules (Chevalier, 2016). Of the metabolism indicators, the Kyn/Trp ratio (marker of systemic IDO activity) and the Cystine/Cys ratio (marker of redox potential) was age‐associated, but the direction of Cystine/Cys ratio was discordant (up in plasma and down in urine), likely a reflection of reduced excretion with age.

In plasma and muscle there were 11 age‐associated metabolites that changed in both compartments in the same direction with only one metabolite (PC ae C34:3) decreasing with age. Choline was one of the top age‐associated metabolites in two compartments. The increase of circulating choline levels in plasma and muscle may be indicative of a healthy population and/or a healthy compensatory increase, as low circulating levels of choline in healthy populations has been associated with poor physical performance (Moretti et al., 2020). Choline is a precursor to PCs, of which 3 PCs (PC aa C38:0, PC aa C40:6, PC ae C34:1) had age‐associated increases in both compartments, with PC aa C36:0 and PC ae C44:5 increasing in muscle and trending up in plasma (*p* = 0.058 and *p* = 0.054, respectively). PCs are formed via choline and the CDP‐choline pathway and betaine via S‐adenosylmethionine (SAM) forming specific fatty acid species of PCs (Zeisel, 2013). Both choline and betaine increased in plasma and muscle, and both are associated with anti‐inflammatory effects and reduced oxidative stress (Mehta et al., 2010; Zhao et al., 2018), although the age‐associated increase could result from reduced renal excretion; however, in our study, neither was age‐associated in urine. Choline and betaine are also precursors for methionine and play a role in circulating levels of sulfur amino acids (Konstantinova et al., 2008). Accordingly, Cys increased with age in plasma and muscle, with homocysteine increasing in plasma. The other changes in the plasma and muscle compartment suggest increased oxidative stress (p‐cresol sulfate; Graboski & Redinbo, 2020) and changes in NO pathways (Arg/SDMA ratio and Orn).

In muscle and urine there were two age‐associated metabolites (GABA and Glu) and levels of both were increased in muscle but decreased in urine, likely resulting from reduced excretion with age. Increased levels of these metabolites in muscle may be beneficial as higher resting muscle Glu levels have been reported in active healthy elderly individuals compared to sedentary healthy individuals (Rutten et al., 2005). The increase may also be resulting from compensatory mechanisms to provide enough energy substrate for muscle (Rutten et al., 2005), or reduced excretion in urine, although, the levels of Glu and GABA were not age‐associated in plasma. This is consistent with the increase of 6 amino acids in muscle (alanine, Arg, Glu, Lys, Cys, Gln) and the decrease of 10 amino acids in urine.

## 3 DISCUSSION

**FIGURE 6:**
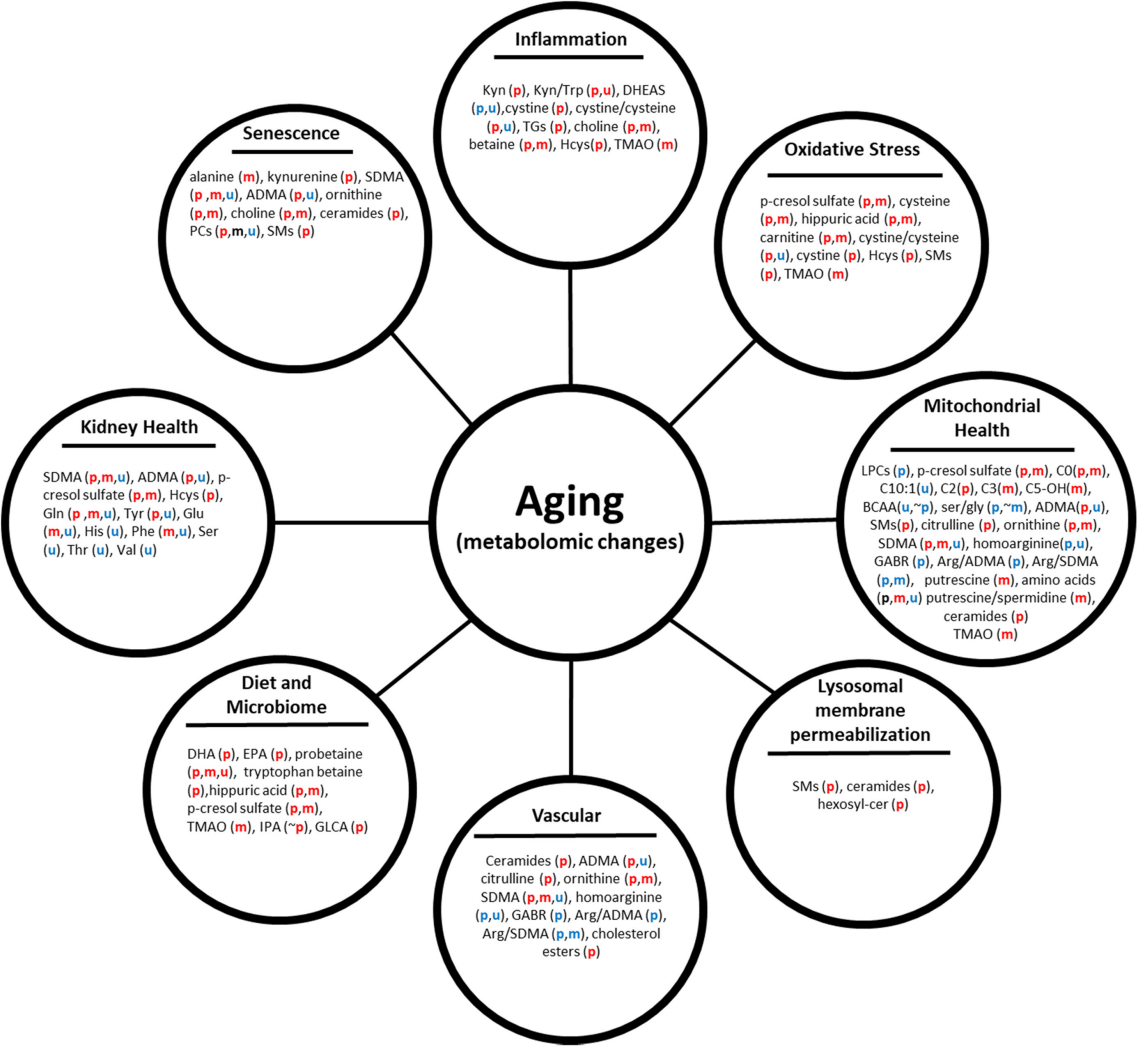
The metabolic pathway that emerged from the metabolomic analysis of plasma (p), muscle (m) and urine (u) from ‘healthy’ participants of the GESTALT cohort (age range 22–93 years). The color indicates direction of the age association (red = increasing with age; blue = decreasing with age; black = mixed directions; ~=trending).

Aging impacts interconnected processes that are reacting and adapting to changes within cells, tissues, and organs (Santos et al., 2019). Some of these processes are reflected in changes in biomarkers in circulation and in tissues. Here, we summarize the metabolic pathways that emerged from our analysis of metabolites in plasma, muscle and urine. These pathways include inflammation and cellular senescence, microbial metabolism, mitochondrial health, sphingolipid metabolism, lysosomal membrane permeabilization, vascular aging, and kidney function (Figure 6; Table S7). It is important to underline that while these biological mechanisms are far from being a comprehensive list of the biological processes at play over human life spans, they provide insight into some of the basic metabolomic age signatures of cross compartmental, interconnected changes.

### 3.1 Chronic inflammation and cellular senescence

Inflammation is a sign of immune activation and is important for defending organisms from bacterial and viral infection but also surveillance of tissue damage as well maintenance, repair and regeneration. Low‐grade chronic inflammation is a feature of aging, and is generally interpreted as a reflection of accumulation of micro injuries and continuous repair (Walker et al., 2022). There is no one definitive source for age‐associated upregulation of inflammation and likely a pro‐inflammatory state with aging is triggered and maintained through multiple mechanisms. These include senescence‐associated secretory proteins (SASPs) secreted from cells undergoing senescence, mitochondrial dysfunction and dysbiosis in the microbiome (Walker et al., 2022). Regardless of the precipitating pathways, there is evidence that inflammation contributes to the development of many age‐related diseases, such as cardiovascular disease, osteoporosis, cognitive impairment, dementia, sarcopenia, and frailty (Sayed et al., 2021). In this study, many metabolites significantly associated with age strongly suggested upregulation of inflammation, including one‐carbon metabolism, oxidative stress, tryptophan metabolism through the kynurenine pathway, reduced arginine bioavailability, declines in mitochondrial health, and increased cellular senescence (Figure 6; Table S7). Age‐associated differences of SASP factors has been previously reported from the plasma and muscle proteome, where 21 and 403 SASP factors were age‐associated, respectively (Johnson et al., 2019; Moretti et al., 2020). In this study, several indicators of an age‐associated increase of cellular senescence was observed including alterations in choline metabolism (Windler et al., 2017), increase in kynurenine (Kondrikov et al., 2020), and perturbed sphingomyelin/ceramide pathways (Trayssac et al., 2018). In addition, several metabolites such as alanine (muscle), kynurenine (plasma), SDMA (plasma, muscle), ADMA (plasma), and ornithine (plasma, muscle) have been identified as SASPs expressed by senescent fibroblasts (James et al., 2015).

### 3.2 Diet and the gut‐microbiome

It has been previously demonstrated that upregulation of the IDO1 expression and activity (Kyn/Trp) (plasma, urine) with age, resulting from lower tryptophan levels (plasma, urine) and/or higher kynurenine levels (plasma), may stimulate the production of pro‐inflammatory cytokines (IL‐1β, IL‐6, IFN‐α, TNF‐α). Indeed, tryptophan plays a central role in the balance between intestinal immune tolerance and gut microbiota maintenance (Gao et al., 2020). Tryptophan is metabolized by gut microbiota generating either indole‐3‐propionic acid (IPA) by *Clostridum Sporogenes* or indole‐3‐aldehyde (I3A) by *Lactobacilli*, and in our study while neither metabolite was significantly age‐associated, there was a trending increase in IPA (*p* = 0.059) with age in plasma. I3A activates the AhR which is a protective factor for the intestinal mucosa and IPA has anti‐inflammatory and antioxidant activity by scavenging hydroxyl radicals and it is a ligand of the pregnane X receptor (PXR) which also has mucosal protective properties (Negatu et al., 2020).

In this study, several metabolites that are associated with dietary intake were positively associated with aging, including proline and tryptophan betaines, Ω‐3 fatty acids (DHA, EPA), and hippuric acid (Figure 6; Table S7). Given that the GESTALT participants were carefully selected to be extremely healthy while dietary assessment was conducted, it is not surprising that especially older participants adhered to a healthy diet. The increase circulating levels of the Ω‐3 fatty acids, is consistent with this hypothesis and confirms a recent prospective cohort study of 2622 community based older US adults, where higher serial levels of EPA, DPA were associated with healthy aging (Lai et al., 2018). Consistent with the idea that older participants adhere to a healthy diet, we observed age‐associated increases of CE of Ω‐3 fatty acids (DHA, DPA, EPA) (Zock et al., 1997). Flavonoids have also been associated with significant health benefits; however, they have poor bioavailability (Bitner et al., 2018). Unabsorbed flavonoids are extensively metabolized by the gut microbiota generating more bioavailable smaller flavonoid microbial metabolites such as valerolactones, phenylalkly acids, and smaller aromatics, including hippuric acid (Bitner et al., 2018). In accordance with our hypothesis of a healthy diet among our older participants, hippuric acid levels were positively associated with age in plasma and muscle. Elevated circulating hippuric acid levels have been associated with greater microbiome diversity and higher intakes of fruit and whole grains in the TwinsUK Study (Pallister et al., 2017), consistent with the healthy status in our cohort. Other metabolites that had age‐associated changes related to the microbiome were p‐cresol sulfate (plasma, muscle), derived from microbial metabolism of tyrosine (Graboski & Redinbo, 2020), TMAO (muscle) derived from dietary choline via the gut flora (Bennett et al., 2013) and IPA (trending) via *Clostridum Sporogenes*. A healthy diet can be very beneficial to human health, not only because of the metabolic substrates provided as energy sources, but because the diet also determines the composition and function of the gut microbiota (Kumar et al., 2016). Additionally, diets considered healthy have been associated with improved mitochondrial health including improved mitochondrial metabolism, mitogenesis, and antioxidant capacity (Khalil et al., 2022).

### 3.3 Mitochondrial health

Mitochondria provide the energy essential for all cellular activities. High availability of energy is essential to counteract the accumulation of damage with aging in nucleic acids, proteins, and lipids (Walker et al., 2022). Mitochondria are a central hub for several age‐associated mechanisms, including inflammation, autophagy, cellular senescence, and genomic stability (Walker et al., 2022). A decline in mitochondrial function with age is probably best studied in an energy demanding tissue, such as the skeletal muscle. Accordingly, the majority of age‐associated metabolites in muscle (amino acids, acyl carnitines, arginine bioavailability, and biogenic amines, PCs) suggest a decline in mitochondrial health with aging (Figure 6; Table S7). Several metabolomic changes were also suggestive of a decline of mitochondrial health with age including carnitine, arginine bioavailability, PCs, ceramides in plasma and/or muscle and BCAA in urine (Figure 6; Table S7). The increase of cystine, Cys, homocysteine, and Cystine/Cys ratio in the plasma compartment and the increase of carnitine and acyl carnitines in both plasma and muscle suggest an increase in ROS, incomplete fatty acid β‐oxidation (Aguer et al., 2015) and therefore impaired skeletal muscle oxidative capacity. The age‐associated increase of *p*‐cresol sulfate in muscle and plasma also support an increased mitochondrial ROS production (Graboski & Redinbo, 2020). The age‐associated decline of BCAA catabolism observed in urine and borderline significance in plasma (*p* = 0.056) may be an indication of a decline in mitochondrial biogenesis and reduced energy production (Esterhuizen et al., 2017). Other indicators of perturbed mitochondrial metabolism, include the decline in the Ser/Gly ratio in plasma and trending decrease in muscle (*p* = 0.059) and age‐associated differences in sphingolipid metabolism. High circulating levels of sphingolipids are associated with inflammation and oxidative stress, and impaired insulin signaling and age‐associated accumulation of ceramide, as observed in plasma, has been shown to result in mitochondrial dysfunction (Li & Kim, 2021).

### 3.4 Lysosomal membrane permeabilization with age

The age‐associated increase of ceramides and SMs in plasma suggest an increase in membrane destabilization with age (Nixon, 2020). Increasing levels of SMs can result in/from an increase in lysosomal membrane permeabilization. Lysosomal membrane permeabilization results in lysosome‐dependent cell death and the release of lysosome contents including cathepsin proteins into the cytoplasm (Wang et al., 2018). Interestingly, an increase of six and two cathepsins was observed in the previously published plasma proteomic data (Tanaka et al., 2018) and skeletal muscle proteomic data (Ubaida‐Mohien, Lyashkov, et al., 2019). HSPA1A which can also contribute to lysosomal stabilization via BMP (Nixon, 2020), was increasing in plasma (Tanaka et al., 2018). BMP is a cofactor that enhances acid sphingomyelinase (ASM) activity, which mediates sphingomyelin catabolism (Nixon, 2020). ASM deficiency can lead to lysosomal membrane permeabilization and cytosolic release of cathepsins (Nixon, 2020). Several additional proteins from the sphingolipid pathway were also age‐associated including the neutral ceramidase (ASAH2, decrease) and LIMP2 (Scarb2, increase) in plasma (Tanaka et al., 2018). The increase circulating hexosylceramides in plasma is consistent with decreased ASAH2 levels with age in plasma (Tanaka et al., 2018).

### 3.5 Vascular aging

Several of the metabolomic differences observed in multiple compartments suggest an aging vasculature including an increase in oxidative stress, decline in arginine bioavailability, decrease in mitochondrial health, lysosomal damage and an increase of inflammation and senescence with age. For example, the increase of Cer(d18:1/16:0) and Cer(d18:1/24:1) in plasma, were previously associated with an increased risk of cardiovascular disease. The most pronounced change across compartments was the decrease in arginine bioavailability that would result in reduced NO synthesis. An increase in nNOS expression in plasma (Tanaka et al., 2018) with a concomitant decrease in arginine bioavailability can result in nNOS uncoupling resulting in the production of superoxide instead of NO (Costa et al., 2016). Impaired NO bioavailability is responsible for age‐related reduction in endothelium‐dependent dilation, enhanced vasoconstriction, and dysregulation of tissue perfusion (Ungvari et al., 2018). With aging, NO production is known to decline and become less effective with accumulation of ADMA, which had an age‐associated increase in plasma. Aging vasculature, which is NO dependent for vasodilation (O'Sullivan et al., 2017), results in a decline in renal blood flow consistent with declining NO synthesis.

### 3.6 Kidney health

Aging is associated with a decline of glomerular filtration rate (GFR) almost without exception in all individuals (O'Sullivan et al., 2017) and is evident in the current study (Figure S5). Decline in GFR is due to the accumulation over time of both anatomic and functional alterations in the kidney on a background of a finite number of nephrons—the functioning units within kidney. A direct consequence of compromised kidney function is lack of excretion (or retention) of endogenous toxins including uremic toxins (ADMA, SDMA), PBUTs (HCys, p‐cresol sulfate), and amino acids. Consistent with this interpretation, several plasma age‐associated plasma proteins reflecting compromised kidney function have been reported (Tanaka et al., 2018) including: CST3, a biomarker of kidney injury/function (Hojs et al., 2006), FGF23 which plays a role in vascular change contributing to the aging phenotype in kidneys (O'Sullivan et al., 2017), and LCN2 and MB which are associated with kidney decline. In the renin angiotensin system (RAS), REN and ACE generate Angiotensin II (Ang II) from Angiotensinogen, and ACE2 catalyzes angiotensin II conversion to angiotensin‐(Cohen et al., 2022; Johnson et al., 2019; O'Sullivan et al., 2017; Tanaka et al., 2018; Teruya et al., 2020; Ubaida‐Mohien, Lyashkov, et al., 2019; Waikar et al., 2010). REN (Tanaka et al., 2018) and ACE2 was measured and ACE2 was not age‐associated in contrast, to a recent study that found an association between lower circulating levels of ACE2 with age (>60 years of age) (AlGhatrif et al., 2021).

## 4 CONCLUSIONS

This study has several strengths including the healthy status of the participants, 2‐year follow‐up measurements of the plasma metabolome in GESTALT, and the validation of the plasma metabolomic data in an independent cohort (BLSA). There are also several limitations in this study, including the generalizability of results to population with different health and demographic characteristics to BLSA and GESTALT studies, cross‐sectional study design, limited sample size, and the limitation of our analyses to plasma, muscle and urine compartments. However, these are the compartments that can be easily accessed in humans, and it would be difficult to have specimens of other compartments in healthy aging individuals. It should also be noted the cross‐sectional design of the study has major limitations, although the design of this study by enrolling ‘healthy’ individuals reduces potential bias dues to chronic diseases having higher prevalence in older persons, it may also be problematic because the ‘healthy’ younger people selected may not remain healthy when they become older (Ubaida‐Mohien et al., 2021). However, longitudinal analysis, while more meaningful, also comes with its limitations including cost‐prohibitive, increase risk of loss of follow‐up (Ubaida‐Mohien et al., 2021) and sample integrity (Wagner‐Golbs et al., 2019). The analysis of a targeted metabolomic approach across multiple compartments offered compartment‐specific as well as compartment‐wide changes with age. Metabolites indicative of chronic inflammation and senescence, one‐carbon metabolism, mitochondrial health and vascular aging were clearly associated with aging in multiple compartments. However, several of these changes were more pronounced in specific compartments, including evidence of an increase in lysosomal membrane permeabilization in plasma, declining mitochondrial function in muscle and declining renal function in urine. While this could simply reflect the concentrations of the associated metabolites in those compartments—for example, the coverage of the SMs and Cers was higher in plasma than in any other compartment—it may indicate that certain compartments are more susceptible to age‐related perturbations of metabolic pathways than others. In addition, there were several age‐associated changes that are consistent with the good health of the participants we studied, including the increase of several metabolites related to diet and beneficial changes related to the microbiome, and the lack of age association of several metabolites and/or metabolism indicators in individual compartments. The results from this study provides insights into important metabolomic aging process that may be used to monitor individuals' health trajectory with aging.

## 5 MATERIALS AND METHODS

### 5.1 Study design and participants

Fasting plasma samples (101) from baseline and from 2‐year follow‐up, muscle biopsies (88) and urine (82) analyzed in this study were collected from participants from the Genetic and Epigenetic Study of Aging and Laboratory Testing (GESTALT). Screening medical exam and blood tests were evaluated to determine whether a candidate participant met the inclusion criteria which were: free of major diseases, except for controlled hypertension or a history of cancer that had been clinically silent for at least 10 years, were not chronically on medications (except one antihypertensive drug), had no physical or cognitive impairments, had a BMI less than 30 kg/m2, and did not train professionally. Inclusion criteria were gathered from information on medical history, physical exams, and blood test interpreted by a trained nurse practitioner at screening and the first study visit (Tanaka et al., 2018; Ubaida‐Mohien, Lyashkov, et al., 2019). Participants were evaluated at the Clinical Research Unit of the National Institute on Aging Intramural Research Program. Whole blood samples were collected using BD vacutainer tubes with ethylenediaminetetraacetic acid (EDTA), then centrifuged at 1231 *g* at 20°C for 15 min; plasma was separated into 500 μL aliquots and stored at −80°C until assay. Participants provided fasting urine (8‐h minimum) samples into a 120 mL sterile urine collection container. Upon collection, the urine was pipetted into 925 μL aliquots. Both plasma and urine aliquots were placed at −80°C until assay.

### 5.2 Muscle biopsy procedure

For baseline visit, muscle tissues were obtained from the vastus lateralis using a 6 mm Bergström biopsy needle. The biopsy site was identified: mid‐point between the great trochanter and the mid‐patella upper margin. The skin was prepped with povidone–iodine (Betadine) and ethyl alcohol, and areas around the biopsy site was covered with sterile drapes. The biopsy site was anesthetized using 1% lidocaine with sodium bicarbonate: intradermally with a 27‐gauge needle, subcutaneously with a 23‐gauge × 1 1/2‐inch needle, and then an 18‐gauge spinal needle to reach the muscle fascia. Special care was used to ensure that lidocaine was infiltrated in the subcutaneous tissue above the muscle fascia but not the muscle fibers so that *no* distortion of the tissue structure and *no* induction of any gene expression responses occurred. After the biopsy site is numbed, a small incision is made in the skin and another incision in the fascia. Then, a 6‐mm Bergström biopsy needle was inserted through the skin opening and then the fascia opening into the muscle. The muscle tissue samples were obtained using a standard method. Biopsy specimens were cut into small sections, snapped frozen in liquid nitrogen, and stored at −80°C until analyses.

### 5.3 Metabolomics panels

Metabolites were extracted in plasma (10 μL) following a previously described protocol (Moaddel et al., 2022). For urine samples (10 μL) were extracted following a similar protocol with slight changes (www.biocrates), including an extended calibration range to account for urine metabolite concentrations, an additional creatinine internal standard was added to account for higher creatinine concentrations with an additional drying step, and a solution of 350 mM urea, 15 mM NaHPO4, pH 6.0 was used as zero samples.

For the skeletal muscle, ~10 mg was homogenized in 0.3 mL tubes from the Precellys Lysing Kit, tissue homogenizing CKMix_WP (Bertin) using 6 μL/mg of 85% ethanol/15% PBS (v/v). The tubes were then homogenized for 3 cycles of 30 s at 5500 rpm with 30 s interval between the homogenization steps using the Precellys Evolution. Afterwards the tubes were centrifuged with a benchtop centrifuge and the solution was transferred to a separate 1.5 mL Eppendorf tube. The tubes were then centrifuged for 5 min at 10,000‐×g in the cold room. Ten microtoliter of supernatant was pipetted onto the center of each well of a 96 well Biocrates kit and the samples dried at room temperature (RT) under nitrogen evaporator for 30 min.

Metabolites were measured using a Nexera HPLC system (Shimadzu) coupled to a 6500 QTRAP® mass spectrometer (AB Sciex) with an electrospray ionization source as previously described (Moaddel et al., 2022). Briefly, the device consists of inserts that have been spiked with internal standards, and a predefined sample amount was added to the inserts. Next, a phenyl isothiocyanate solution was added to derivatize some of the analytes (e.g. amino acids), and after the derivatization was completed, the target analytes were extracted with an organic solvent, followed by a dilution step and samples were analyzed by flow injection analysis‐tandem mass spectrometry (FIA‐MS/MS) and liquid chromatography–tandem mass spectrometry (LC–MS/MS). Briefly, two UHPLC methods were run using the MxP Quant 500 Column System for the LC–MS/MS methods with the mobile phase consisting of solvent A (water containing 0.2% formic acid) and solvent B (acetonitrile containing 0.2% formic acid). For the FIA‐MS/MS method, the FIA plate was run at a flow rate of 30 μL/min with FIA solvent as the mobile phase. Concentrations were calculated using the MetIDQ software and reported in μmol/L. Data were quantified using appropriate mass spectrometry software (Sciex Analyst®) and imported into Biocrates MetIDQ™ software for further analysis. The data was normalized to internal quality controls. Pooled samples were repeated across plates to assure for accuracy of the measurements across plates. The kit potentially measures 630 metabolites, but 164 metabolites in plasma and 463 metabolites in muscle and 490 metabolites in urine were either completely below the limit of detection or were excluded from the analysis because they were below the limit of detection in more than 30% of subjects. After exclusions, 435 metabolites (plasma) 144 metabolites (skeletal muscle) and 93 metabolites (urine) were available for the data analysis. By choosing the same platform for all compartments, we reduced variability that can result from different chromatographic methods. Based upon metabolite concentrations, additional ratios (metabolism indicators) were calculated to aid the interpretation of pathways (Table S6).

### 5.4 Data processing, statistical analysis, and visualization

Metabolites with >30% missing samples were excluded from analysis and the missing data is further analyzed to make sure the missingness is random across all age groups and not because the metabolites are not detectable of aging process (metabolites were not detectable at a young age but increased with aging or vice versa). Metabolites with <30% missing values were imputed by 1/5 of minimum positive values of each variable. Each metabolite abundance was log transformed and the metabolite concentrations were used to build the 3D principal component analysis (PCA). PCA was used for multivariate statistics, specifically for participant potential outlier detection, assessment of compartment differences and visualization of multiple compartments.

The outlier samples (>30% missing metabolites for a sample) and LOD metabolites were removed, thus the final number of participants sample include 101 participants (plasma at baseline), 65 participants at visit 2, skeletal muscle metabolites sample from 88 participants, and urine metabolites from 82 participants. Of the 630 metabolites measured for each compartment, 435, 144, and 93 metabolites were quantitative, respectively.

Each metabolite was analyzed for age association by a multivariable linear regression model adjusted for sex, race and BMI using the lm() function from the base R package stats (v. 3.6.1). To account for multiple comparisons, *p*‐values were corrected by the Benjamini–Hochberg procedure to control the false discovery rate and reported in the Table S3. The threshold for statistical significance was *p*‐value <0.05 unless otherwise reported for metabolites. Calculated *p*‐values for age association and effect size (β_age_ or log2 fold‐change per year) are indicated in the figure panels or legends. All statistical analysis were conducted using R (3.6.1) with built‐in libraries and functions. The PCA plot, volcano plot and heat map to visualize metabolite‐age associations results were generated using ggplot2 function, Enhanced Volcano plot, ComplexHeatmap, data analysis tool extension R libraries, and GraphPad Prism (9.3.0). Spearman correlation was used to assess the relationship between beta estimates of age and plasma metabolite associations at baseline and 2‐year follow‐up, and between GESTALT and BLSA metabolites. Pathway information was determined with extensive literature research for clinical and functional aging analysis. Gene Ontology enrichment analysis was performed by ClueGO, pathway analysis was performed by String and Reactome database. Pathway visualizations were performed in Cytoscape (3.8.0).

### 5.5 Data validation

To validate findings on metabolite‐age association from the GESTALT study, plasma metabolomic data from 162 participants from the Baltimore Longitudinal Study of Aging (BLSA; https://www.blsa.nih.gov/) were used. The inclusion criteria for the subset of BLSA samples were comparable to the GESTALT at entry. The age range of the BLSA participants were between 22 and 97 years. The method for plasma metabolite assessment and data processing was consistent with GESTALT procedure. A subsample of 162 BLSA participants who met IDEAL status shared similar health characteristics as the GESTALT study participants. Specifically, since 2007 at the BLSA study enrollment, highly standardized criteria for excellent or ‘IDEAL’ (Insight into the Determinants of Exceptional Aging and Longevity) health have been established based on physical and cognitive function, absence of major diseases (except controlled hypertension and cancer clinically silent for over 10 years), and laboratory blood values (Ferrucci, 2008; Schrack et al., 2014). ‘IDEAL’ status is adjudicated weekly by the BLSA research team based on pertinent data collected on each BLSA participant.

## AUTHOR CONTRIBUTIONS

RM and LF conceived and designed the experiment. MK, GF, NS, VC, LT, and CWC were involved in acquisition and handling of clinical samples. JL carried out the acquisition of the data for GESTALT studies and QT provided data for BLSA studies. CU and RM carried out data analysis. RM, CU, JME, LF carried out interpretation of the data. RM drafted the original manuscript and all authors critically reviewed the manuscript and contributed to the final manuscript. All authors agree to be accountable for all aspects of the work.

## CONFLICT OF INTEREST STATEMENT

None declared.

## Supporting information


Data S1

Data S2

## Data Availability

Individual participant data that underlie the results reported in this article, after de‐identification (text, tables, figures, and appendices) will be available upon request. Documents of the statistical analysis plan and analytic code will be available upon request. Scientists interested in accessing the data should access the website https://www.blsa.nih.gov/ and submit a methodologically sound proposal to the BLSA committee.
