# Supplementary material for: Cross‐sectional analysis of healthy individuals across decades: Aging signatures across multiple physiological compartments
Source: Aging Cell. 2023 Jun 23;23(1):e13902. doi: 10.1111/acel.13902 (PMC10776121; doi:10.1111/acel.13902)
Supplement: Supplementary file 2 — Data S2 [file ACEL-23-e13902-s001.pdf]

Supplementary Figures

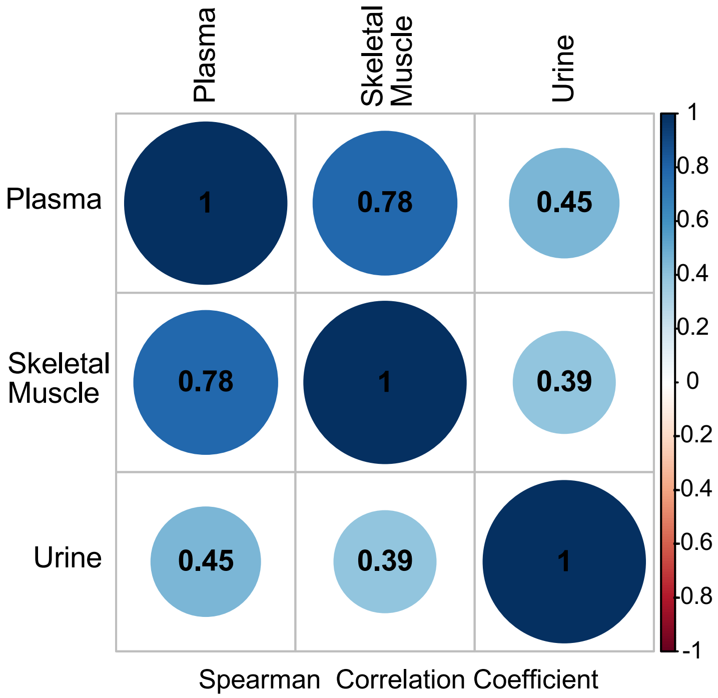

Supplementary Figure 1: Correlation between plasma, skeletal muscle, and urine compartments. Common metabolites (log2 concentration) across all three compartments were used for the analysis. For each metabolite, the average metabolite concentration of all participants was calculated for each compartment. The Spearman correlation coefficient is shown on the right.

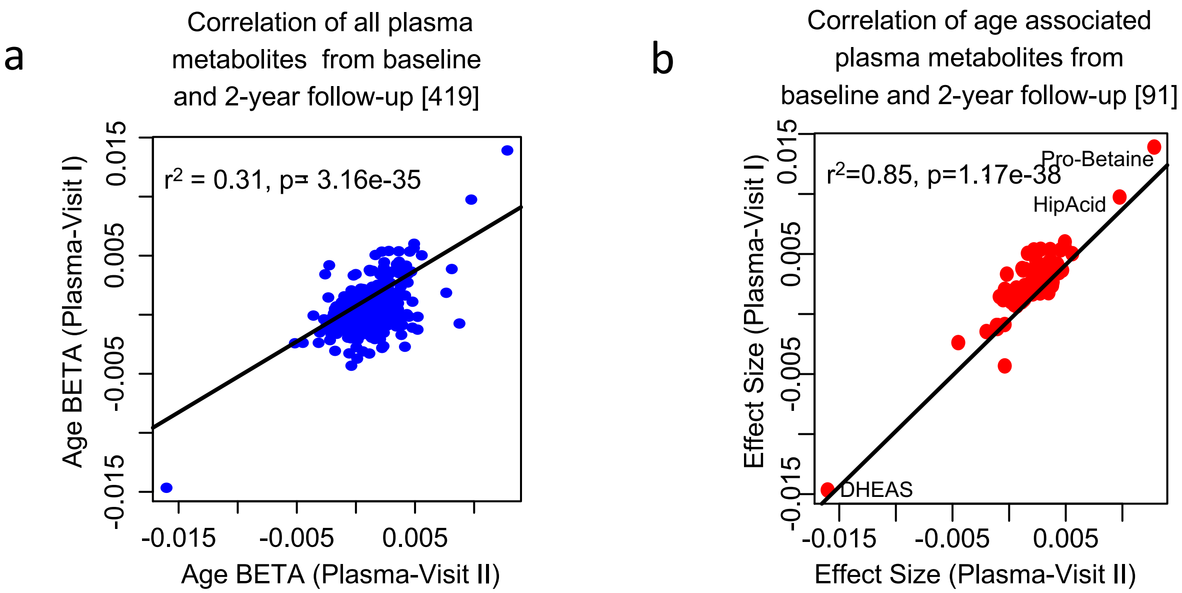

Supplementary Figure 2: Age beta correlation of plasma metabolites from baseline and 2-year follow-up. **a)** All 419 plasma metabolites from baseline and the 2-year follow-up are correlated to each other. **b)** 91 age-associated metabolites from baseline are correlated to 2-year follow-up, r2, p-value and top correlated metabolites are plotted.

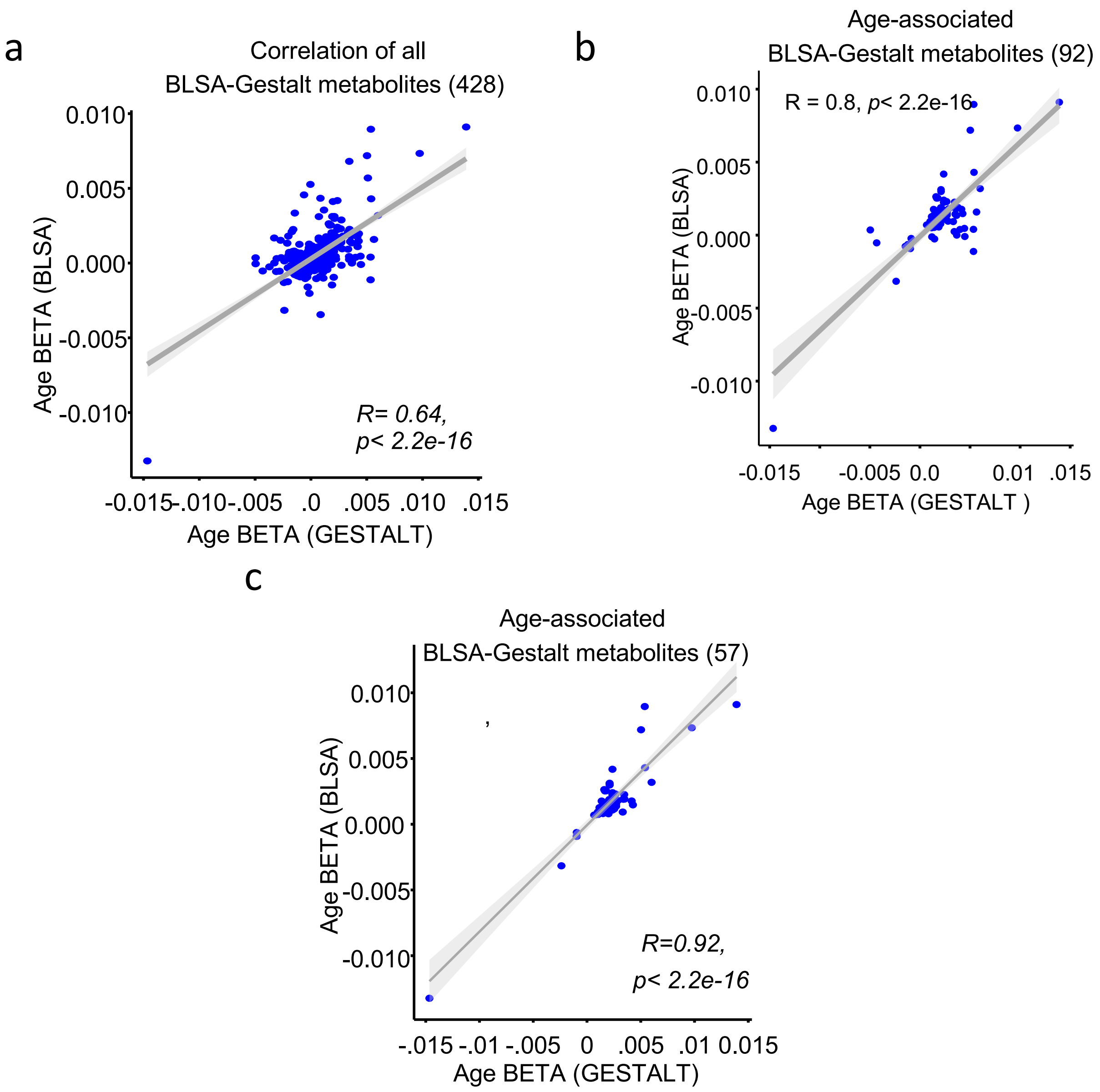

Supplementary Figure 3: GESTALT plasma metabolite validation using an independent healthy aging cohort (BLSA). a) Correlation of 428 BLSA and GESTALT plasma metabolites. Age beta (age-effect size) is correlated between two datasets using Pearson correlation. **b)** Age beta (age-effect size) of 92 significant age-associated plasma metabolites ($p<0.05$) from GESTALT is correlated with BLSA data. **c)** 57 common age-associated GESTALT and BLSA metabolites correlation. The correlation coefficient is measured by Pearson correlation and the p-value is plotted on right.

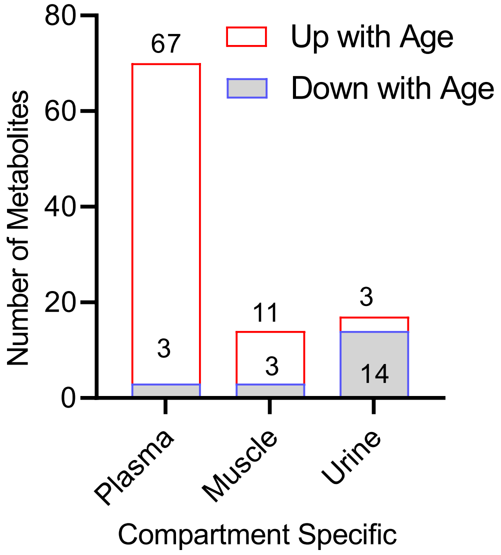

Supplementary Figure 4: Compartment specific changes in plasma, muscle and urine. The number of metabolite increase or decrease with age for each specific compartment is shown on the bar plot.

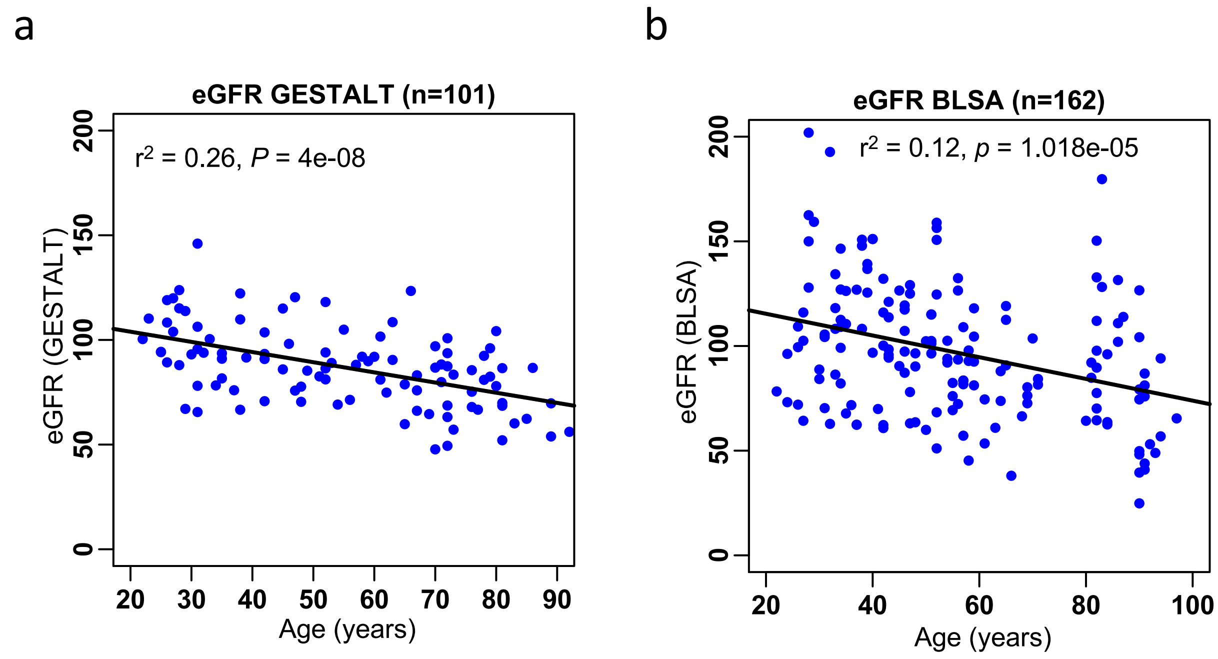

Supplementary Figure 5: eGFR for GESTALT and BLSA cohort. a) GESTALT eGFR. **b)** BLSA eGFR**.** eGFR is on y-axis and the age of the participants is on the x-axis, each blue circle is a participant.
